# Differential impacts of jogging and rope skipping in college students in China based on physical test score: a randomized controlled trial baseline indicator comparison in the intervention

**DOI:** 10.3389/fpubh.2025.1570768

**Published:** 2025-04-08

**Authors:** Yuxuan Chen, Junjie Wu, Zhuo Xu, Rucheng Chen, Qinghua Sun

**Affiliations:** School of Public Health, Zhejiang Chinese Medical University, Hangzhou, China

**Keywords:** physical activity, jogging, rope skipping, college student, physical fitness

## Abstract

**Introduction:**

Studying the differential effects of jogging and rope skipping provides useful insights for teens, adolescents, and even adults to make choices and maintain healthy physical activities, which may also have positive significance for the promotion of public physical fitness and health.

**Methods:**

A total of 92 college students were enrolled in the study and randomly divided into jogging group, rope skipping group and control group. Tests and questionnaires were conducted before and right after the intervention, and a questionnaire survey was conducted again half a year after the intervention.

**Results:**

The results showed that the standing long jump performance and the cardiopulmonary function in the jogging group were significantly improved after the intervention, the average score of standing long jump increased from 72.00 to 76.45 and the mean systolic blood pressure decreased from 125.07 to 121.24 (*t* = 3.048, 2.139, *p* = 0.005, 0.041). The scores of 800 or 1,000 meters and the total scores of physical test in the rope skipping group and the control group were decreased significantly. The improvement of standing long jump performance in the jogging group was significantly higher than that in the control group.

**Discussion:**

The main reason for the decline in the rope skipping group may be due to insufficient strength. Jogging differs from rope skipping in terms of muscle focus, arm-swinging style, nature of the exercise, and the associated exercise mood. As a result, after short-term training, jogging has a more positive significance than skipping rope in maintaining and improving physical fitness and cardiopulmonary function. If you want to improve your physical fitness in a shorter period of time, then jog.

## 1 Introduction

Chronic disease is a major public health challenge globally (1), and the incidence tends to be younger in recent years (2). Lifestyle, especially physical activity, is closely related to major chronic diseases (3). According to previous study, the vast majority of deaths in the world are caused by chronic non-communicable diseases, mainly cardiovascular and respiratory diseases (4). Physical activity has been demonstrated to reduce the risk factor of chronic non-communicable diseases in a dose-dependent manner (5) and promotes general health (6). On the contrary, lack of physical activities enhances cardiovascular risk (7), increases the risk of other chronic non-communicable diseases, and greatly increases the morbidity and mortality from these diseases (8), which results in substantial decreases in both total and quality years of life (9).

Jogging and rope skipping are regarded as two of the most common physical activities. They both are relatively easy to perform, with high popularity and acceptance in general population, which have minimum requirement and few restrictions in terms of time and place. In simple terms, both are very accessible and easy to be implemented on campus (10, 11). Jogging, with its strong mass base in the current era of national fitness, offers several advantages such as easy, popular, low-cost, suitable for many circumstances, and significant fitness benefits (12). On the other hand, rope skipping, a traditional sport with a long history and similar advantages with jogging, has been well promoted and popularized in the teens and adolescents in China in recent years (13). Studies have shown that jogging combined with rope skipping has a significant effect on reducing body weight, and plays a positive role in improving and maintaining body shape, body function, and body self-esteem (14). However, there is a noticeable gap in the existing literature that no study has tested the differential impact of regular jogging and rope skipping on enhancing the physical health of college students, which may be important and helpful in physical activity promotion and practice.

In recent years, with the development of society, people’s lifestyles have also changed that is characterized by an evident lack of physical activity, which has become a global problem (15) that becomes the fourth leading cause of death worldwide (16). Thus, enhancing the level of physical activities has become one of the top priorities in public health policies in numerous countries globally. The World Health Organization (WHO) indicates that one in five adults, and four out of five adolescents (11–17 years), do not have enough physical activity worldwide (17), and recommends that individuals should maintain sufficient exercise and physical activity patterns can be changed through effective interventions (18). In addition, one study found that about half of young adults (18–29 years old) who were enrolled in colleges did not meet the recommendations for physical activity (19). And a significant proportion of college students engaged in higher levels of sedentary time compared to the general young population, and their accumulated levels of sedentary time was associated with an increased risk of adverse health outcomes (20). Furthermore, according to the relevant requirements of the “Healthy China 2030 Plan” and the “Medium-and Long-Term Youth Development Plan (2016–2025),” China has implemented the national strategy of physical fitness and proposed the execution of extensive national fitness activities, the reinforcement of youth sports initiatives, the advancement of mass sports and competitive sports for comprehensive development, and the acceleration of becoming a sports power in the world (21, 22).

At the same time, lifestyle, especially physical activity, is developed overtime. The earlier it starts to form a healthy lifestyle with routine physical activity, the more likely it lasts longer, if not lifetime. When one is young, it is more important to cultivate positive physical activity identity (23). Research also indicates that physical education in elementary and secondary schools plays a crucial role in nurturing students’ habits and spirits for physical activity, which are essential for adhering to lifelong physical activities in the future (24). It is critical and timely to focus on healthy life and disease prevention in adolescence in which physical activity should be included (25). Therefore, cultivating the interests and habits of physical activities during schooltime has a profound impact on the formation of a long-term and sustainable healthy lifestyle.

Therefore, we recruited college students to carry out exercise intervention of jogging or rope skipping with health physical education guidance, aimed to explore their differential effects on physical and functional tests, and hoped to provide useful insights for the teens, adolescents and even adults to make choices and maintain healthy physical activities, which may also have positive significance for the promotion of public physical fitness and health.

## 2 Methods

### 2.1 Study design

College students at Zhejiang Chinese Medical University in the City of Hangzhou, Zhejiang Province of China were recruited as the research subjects. Simple randomization was used, and Excel was utilized to generate random numbers. The participants were ranked based on the order of recruitment and then randomized into jogging group, rope skipping group or control group via random numbers. During the intervention, a software named Keep was used for the management and supervision of the intervention, which required a collection of motion screenshots to be submitted once a week, and field spot checks were performed periodically. Before and right after the interventions, all participants were evaluated for physical fitness, body composition and cardiopulmonary function. An electronic stopwatch (Jinque, Model JD-3BII, Shanghai, China), a high-precision electronic vital capacity tester (Wanqing, Model WQS-8888, Measuring range 10,000 mL, Indexing value 1 mL, Accuracy ± 1.5%; Shanghai, China) and an electronic sphygmomanometer (Cofoe, Model KF-65A, arm type; Changsha, Hunan Province, China) were used to test physical fitness and cardiopulmonary function. A human body composition analyzer (Donghuayuan, Model BC210, measurement frequency 5 kHz, 50 kHz, 250 kHz; Changping, Beijing Province, China) was used to evaluate body composition. Two testers of primary and secondary were assigned to record the test data. The data from the primary one were adopted if the data from these two were similar. Tests would be redone and the data from these two were averaged for recording if the difference was big. And testers were blinded to avoid bias. Additionally, a questionnaire survey that included some of the International Physical Activity Questionnaire Short Form, the Global Physical Activity Questionnaire, and “WHO 2020 guidelines on physical activity and sedentary behavior” (26) was conducted before, right after, and half a year later after the exercise intervention. The participants in the control group maintained their physical activity without intentional or significant change during the study, and physical tests and functional evaluation along with questionnaire were conducted the same as in the intervention groups. The study was reviewed and approved by the Medical Ethics Committee of Zhejiang Chinese Medical University, 20221011-3, 11 October, 2022 and is registered with the clinical trials registry (http://clinicaltrials.gov, ID: NCT05791500, 30/03/2023). All research was performed in accordance with relevant guidelines/regulations. The study procedure is illustrated in Supplementary Figure S1.

### 2.2 Study participants

The inclusion and exclusion criteria of participant recruitment were as follows: (1) college current undergraduate students regardless of gender; (2) For your health and safety, and for unaffected performance evaluation, no major disease history, or no major trauma or surgery history in the past 2 years; (3) no any special treatment or medication or being in a state of intervention; (4) can participate and complete the study requirements with high quality. The required sample size was estimated to be 91, which was based on a two-sided z-test with a significance level of 5%, a power of 0.9, and an estimated difference of two. The calculation formula is shown in equation (1). We recruit participants by setting up billboards on the university campus and students registering voluntarily. After initial screening, a total of 100 college students from Zhejiang Chinese Medical University were recruited in the study and were randomized into one of the three groups: jogging group, rope skipping group and control group. During the study, eight participants dropped out in the mid of the study (one of illness and seven of interest lost), including four in the jogging group and four in the rope skipping group. In total, 92 participants (29 in the jogging group, 29 in the rope skipping group, and 34 in the control group) successfully completed the study. The selection process of RCT is shown in Supplementary Figure S2. The basic characteristics of the participants are shown in Table 1. All participants were informed in advance of the study purpose, methods, and potential risks, and written informed consents from them were obtained.

**Table 1:** Participants’ characteristics and baseline indicator comparison in the intervention study.

Item	Control group (*n* = 34)	Jogging group (*n* = 29)	Rope skipping group (*n* = 29)	Total (*n* = 92)	Statistics (χ^2^/F/H)	*p*	η^2^
Sex—no. (%)							
Male	9 (26.5)	10 (31.0)	10 (34.5)	29 (30.4)	0.482	0.812	0.005
Female	25 (73.5)	20 (69.0)	19 (65.5)	64 (69.6)			
College year—no. (%)							
Freshman	10 (29.4)	7 (24.1)	9 (31.0)	26 (28.3)	2.475	0.896	0.008
Sophomore	11 (32.4)	9 (31.0)	6 (20.7)	26 (28.3)			
Junior	13 (38.2)	12 (41.4)	13 (44.8)	38 (41.3)			
Senior and above	0	1 (3.4)	1 (3.4)	2 (2.2)			
College/School name—no. (%)							
First Clinical Medical College	9 (26.5)	13 (44.8)	7 (24.1)	29 (31.5)	/	0.608	0.014
Second Clinical Medical College	4 (11.8)	2 (6.9)	2 (6.9)	8 (8.7)			
Third Clinical Medical College	3 (8.8)	1 (3.4)	5 (17.2)	9 (9.8)			
Fourth Clinical Medical College	2 (5.9)	0	1 (3.4)	3 (3.3)			
School of Public Health	10 (29.4)	8 (27.6)	9 (31.0)	27 (29.3)			
College of Nursing	1 (2.9)	2 (6.9)	2 (6.9)	5 (5.4)			
College of Life Sciences	3 (8.8)	2 (6.9)	0	5 (5.4)			
College of Pharmacy	0	1 (3.4)	1 (3.4)	2 (2.2)			
College of Medical Technology and Information Engineering	2 (5.9)	0	2 (6.9)	4 (4.3)			
Body composition score—mean ± SD	73.31 ± 5.53	71.51 ± 5.06	71.75 ± 4.05	/	1.245	0.293	0.027
Total score of physical test—mean ± SD	79.95 ± 9.74	78.80 ± 8.11	78.07 ± 7.78	/	0.380	0.658	0.008
BMI score—medians (IQR)	100.00 (20.00)	100.00 (0.00)	100.00 (0.00)	/	3.162	0.206	0.032
Sit-ups (female) or pull-ups (male) score—medians (IQR)	72.00 (47.50)	72.00 (29.00)	78.00 (57.50)	/	0.590	0.744	0.004
Standing long jump score—mean ± SD or medians (IQR)	74.76 ± 13.35	72.00 (14.32)	72.00 (14.00)	/	0.879	0.644	0.018
Sitting forward flexion score—medians (IQR)	80.15 (10.60)	85.00 (16.50)	78.00 (20.00)	/	1.425	0.490	<0.001
Vital capacity score—medians (IQR)	100.00 (15.00)	85.00 (22.00)	85.00 (23.00)	/	4.415	0.110	0.024
50 meters score—mean ± SD or medians (IQR)	74.73 ± 6.49	74.00 (13.00)	75.79 ± 9.21	/	0.210	0.900	0.007
800 (female) or 1,000 (male) m score—mean ± SD or medians (IQR)	76.00 (15.00)	74.10 ± 10.31	76.00 (14.00)	/	3.356	0.187	0.062
Systolic blood pressure (mmHg)—mean ± SD	121.24 ± 11.84	125.07 ± 10.56	125.07 ± 9.43	/	1.370	0.260	0.030
Diastolic blood pressure (mmHg)—mean ± SD	65.65 ± 8.21	67.66 ± 7.61	68.17 ± 6.79	/	0.987	0.377	0.022
Heart rate (beat/min)—mean ± SD	79.32 ± 14.59	79.03 ± 9.65	84.07 ± 15.31	/	1.302	0.277	0.028

Numbers in parentheses in the participant characteristics indicate the percentage of this population in the group; MBI score, sit-ups (female)/pull-ups (male) score, standing long jump score, sitting forward flexion score, vital capacity score, 50 meters score and 800 (female)/1,000 (male) meters score are calculated based on “National Student Physical Health Standard” (27), respectively.

### 2.3 Interventions

From March to May, 2023 of 2 months, the participants in the two intervention groups were trained in the jogging and rope skipping, respectively, at least four times a week, at least 50 min each time for the males and at least 40 min each time for the females. The participants in the control group maintained their previous intensity of physical activity without intentional or significant change. The details are shown in Supplementary Table S1.

A physical activity teacher who was a professional athlete with rich experience in physical education and recently retired from China national team led the physical education and guidance to the intervention groups before the exercise training intervention in order to ensure the quality of training during the study period and prevent possible injuries.

### 2.4 Outcomes

The leading outcome of this study was the total scores of physical test, which was used to evaluate the physical quality of the participants. The followings were secondary outcomes. According to the “National Student Physical Health Standard” (27), the physical test scores included college students’ body mass index (BMI), sit-and-reach, standing long jump, sit-ups (female) or pull-ups (male), 800 (female) or 1,000 (male) meters run, 50-meters run to evaluate students’ flexibility, explosive power and endurance. The bc210 body tester was used to analyze body composition, and the participants’ physical quality was evaluated by scoring. Systolic and diastolic blood pressures, heart rate and other indicators were selected to evaluate their cardiopulmonary function. The questionnaires were scored to analyze the changes of the participants’ physical activities, which might reflect the development of their habits.

### 2.5 Data analysis

Categorical variables are presented as a sample percentage (%), and continuous variables are presented as means with standard deviation (SD) or medians with interquartile range (IQR) for the variables with normal or non-normal distribution, respectively. Prior statistical analysis, Shapiro–Wilk test was used to verify the normality of variables distribution. Subsequently, Chi-square test or Fisher exact probability method were used to analyze the differences between groups of the categorical variables. T-test or Wilcoxon test were applied to analyze the differences between two groups of the continuous variables. ANOVA analysis and Kruskal-Wallis test were used to analyze the differences among multiple groups of the continuous variables. The test level was two-sided *α* = 0.05. All statistical analyses were performed using SPSS25.0 software, and variables with a significance level below 0.05 were deemed statistically significant. And understand the accuracy of the estimation through the confidence interval, and learn about the effect size of the independent variable on dependent variable through eta-squared.

We calculated the differences of physical test scores, body composition scores and cardiopulmonary function indexes before and after the intervention in each group (jogging group, rope skipping group and control group), and analyzed whether the intervention was effective. The differences in physical test scores, body composition scores and cardiopulmonary function indexes between groups before and after the intervention were analyzed to see whether there were differences in the intervention effects from different physical activities. The questionnaire scores of each group before, right after and half a year after the intervention were analyzed to explore the differences between different interventions on the development of physical activity habits. Additionally, sensitivity analysis was performed on the leading outcomes to assess the robustness of the research methods and results.

## 3 Results

### 3.1 Baseline data analysis of study participants

The characteristics including sex, college year, and names of college or school of the participants among jogging group, rope skipping group and control group before the intervention were collected, and their physical test scores, body composition scores and cardiopulmonary function indexes were evaluated. The results showed that there was no significant difference in baseline data among the three groups, which are shown in Table 1.

### 3.2 Total score of physical test

The differences of physical test scores before and after the intervention in jogging group, rope skipping group and control group were analyzed. The results showed that before and after the intervention, the total scores of the physical test in the rope skipping group and the control group were statistically significant (*p* < 0.05). Among them, the total scores of the physical test of the rope skipping group were decreased significantly after the intervention (*t* = −2.130, *p* = 0.042), and the total scores of the physical test of the control group were decreased significantly (*t* = −2.164, *p* = 0.038). However, the difference analysis of the total physical test scores of the jogging group, the rope skipping group and the control group after the intervention showed that there was no significant difference in the total physical test scores of the three groups (*F* = 0.473, *p* = 0.625). The details are shown in Table 2.

**Table 2:** Comparison of total physical test scores between groups before and after the intervention.

Group	Control group (*n* = 34)	Jogging group (*n* = 29)	Rope skipping group (*n* = 29)	Statistics (*F/H*)	*p*	η^2^
Prior intervention	79.95 ± 9.74	78.80 ± 8.11	78.07 ± 7.78	0.380	0.658	0.008
Post intervention	78.12 ± 10.28	78.27 ± 6.60	76.25 ± 9.13	0.473	0.625	0.011
d	−1.83 ± 4.94	−0.54 ± 4.45	−1.42 ± 4.21	0.172	0.842	0.018
95%CI	[−3.55, −0.11]	[−2.23, 1.16]	[−3.57, −0.07]	/	/	/
Statistics(*t*)	−2.164	−0.648	−2.130	/	/	/
*p*	0.038	0.522	0.042	/	/	/

d, the data after the intervention minus the ones before the intervention.

### 3.3 Other outcomes

The analysis of physical test scores and body composition scores showed that before and after the intervention, the standing long jump of the jogging group, the 800 or 1,000 meters of the rope skipping group, and the 800 or 1,000 meters of the control group were statistically significant (*p* < 0.05). Among the above indicators, compared with the ones before the intervention, the standing long jump performance of the jogging group was improved significantly after the intervention (*t* = 3.048, *p* = 0.005); the 800 or 1,000 meters scores of the rope skipping group were decreased significantly (*t* = − 2.521, *p* = 0.012); the 800 or 1,000 meters scores of the control group were decreased significantly (*Z* = 2.983, *p* = 0.003). The detailed results are shown in Supplementary Table S2. However, the difference analysis of the secondary outcomes of the jogging group, the rope skipping group and the control group after the intervention showed that there was no significant difference of the three groups (*F* = 0.473, *p* = 0.625). The details are shown in Table 3.

**Table 3:** Comparison of secondary outcomes between groups after the intervention.

Parameter	Control group (*n* = 34)	Jogging group (*n* = 29)	Rope skipping group (*n* = 29)	Statistics (*F/H*)	*p*	η^2^
Body composition score	70.75 (5.10)	71.74 ± 4.86	70.80 (6.20)	0.523	0.770	0.008
BMI score	100.00 (20.00)	100.00 (0.00)	100.00 (0.00)	1.747	0.417	0.028
Sit-ups (female) or pull-ups (male) score	70.00 (47.00)	70.00 (21.00)	72.00 (27.50)	0.917	0.632	0.002
Standing long jump score	74.00 ± 15.46	76.45 ± 11.92	72.00 (13.00)	1.518	0.468	0.035
Sitting forward flexion score	78.15 ± 11.08	80.31 ± 10.39	78.00 ± 10.86	0.425	0.655	0.009
Vital capacity score	90.00 (20.00)	90.00 (25.00)	80.00 (19.00)	2.725	0.256	0.046
50 meters score	74.56 ± 10.15	72.00 (8.00)	76.00 (8.50)	1.751	0.417	0.015
800 (female) or 1,000 (male) meters score	73.47 ± 15.57	72.28 ± 11.51	72.00 (12.50)	1.569	0.456	0.029
Systolic blood pressure (mmHg)	119.29 ± 11.84	121.24 ± 10.07	128.00 (20.00)	5.493	0.064	0.065
Diastolic blood pressure (mmHg)	65.91 ± 9.29	66.93 ± 8.66	67.10 ± 7.54	0.181	0.835	0.004
Heart rate (beat/min)	81.00 ± 14.22	80.14 ± 11.07	82.21 ± 13.05	0.188	0.829	0.004

The analysis of cardiopulmonary function indexes showed that there was a statistically significant difference between systolic blood pressure before and after the intervention in the jogging group. Systolic blood pressure after the intervention was significantly lower than that before the intervention (*t* = 2.139, *p* = 0.041), which are detailed in Supplementary Table S2.

The results also showed that the *d* values (the data after the intervention minus the ones before the intervention) of standing long jump scores before and after the intervention among the three groups were statistically significant (*H* = 6.247, *p* = 0.044). The improvement of standing long jump performance in the jogging group was significantly higher than that in the control group (*Z* = −2.460, *p* = 0.014). See Supplementary Tables S3, S4 for the details.

The analysis of questionnaire scores showed that there was no significant difference half a year after the intervention among the three groups, and there was also no statistical difference in the *d* values (the questionnaire scores half a year after intervention minus the ones before the intervention) among the three groups. The details are shown in Supplementary Table S5.

### 3.4 Sensitivity analyses for the leading outcome

In this study, the gender-stratified analysis of physical test total score as the leading outcome was performed, and the comparisons among the groups did not change significantly. The details are shown in Supplementary Table S6. In addition, we used three statistical models to analyze the effects of different physical activity interventions on total physical test scores according to the division of different subgroups, and the significance of the difference did not change substantially (Supplementary Table S7).

## 4 Discussion

Designed and aimed to explore the differential impacts of jogging and rope skipping on physical activity and fitness in college students primarily across medicine-related majors, there are several major findings in this interventional trial study. First, standing long jump performance in the jogging group was significantly improved after the intervention. Second, cardiopulmonary function in the jogging group was significantly improved after the intervention. Third, 800 or 1,000 meters scores in the rope skipping group were significantly decreased after the intervention. Fourth, total scores of physical test in the rope skipping group were significantly decreased after the intervention. Fifth, the improvement of standing long jump performance in the jogging group was significantly higher than that in the control group. In addition, there was no significant difference in the total scores of physical test between the groups after the intervention. Furthermore, their physical activity returned to its original level half a year after the intervention.

Physical activity is of great significance for effective physical fitness enhancement. Through a period of regular physical activities, some indexes of college students’ physical quality were significantly improved for a short-term. Jogging and rope skipping are two different sport activities to improve physical quality and cardiopulmonary function with different focuses. Studies have shown that the quality of physical fitness is usually related to genetic factors, acquired nutrition and physical activities. To our knowledge, previous studies have not or rarely examined the differential effects on physical fitness between jogging and rope skipping in college students. Some study about the respective roles of jogging and rope skipping found that jogging could improve cardiovascular and respiratory functions, promote human health, in order to enhance physical fitness (28), while rope skipping could enhance muscle strength, flexibility, coordination and durability, and improve the level of physical fitness (29–31). The study of Baker (32) found that 10 min of rope skipping and 30 min of jogging per day can significantly improve cardiovascular efficiency; it is believed that 10 min per day rope skipping and 30 min per day jogging are equally effective in improving cardiovascular efficiency. However, Buyze et al. found that the maximum increase in oxygen content in the people who performed jogging intervention was greater than that in the people who performed rope skipping through a six-week intervention plan. Therefore, they believe that 10-min skipping would not cause training response comparable to 30-min jogging (33). This is consistent with our findings to some extent, but the intensity and duration of the exercises in our study are different from theirs. In our study, the standing long jump performance of the jogging group was significantly improved after the intervention, while the 800 or 1,000 meters scores and the total scores of the physical test in the control group were significantly decreased. At the same time, the jogging group’s standing long jump performance progress was significantly higher than that of the control group. It demonstrates that short-term jogging intervention plays a positive role in enhancing physical health. Nevertheless, the 800 or 1,000 meters scores and the total scores of the physical test in the rope skipping group were significantly decreased. One study has shown that progressive rope skipping for 50 min each time, three times a week for 8 weeks may improve physical health and promote cardiovascular health (34). Another study has also shown that 12 weeks of rope skipping 2,000 jumps daily can reduce cardiac and metabolic risk factors (35). To our study, we believe that there might be a few factors that cause the differences, which were primarily in our study design that the intensity, number and single duration of rope skipping were different or too low. The requirements in our study included only the minimum standards, and most participants only completed the minimum requirements. These standards did not seem to impact effectively or substantially in improving physical fitness. At the same time, with this relatively short intervention time period, it could not achieve the goal of enhanced physical fitness. In the future research, we have a plan that includes an improved design in the intensity, number and exercise duration of rope skipping in hope to achieve better and long-lasting effects.

Physical activity also has obvious significance in improving cardiopulmonary function. Heart rate and blood pressure are two important indicators for cardiopulmonary function evaluation (36, 37). Active and regular exercise can reduce the risk of hypertension and improve physical fitness and health. Regular (> 3 days per week), moderate intensity exercise for each duration (30–45 min or more) can reduce systolic blood pressure by 5–17 mmHg and diastolic blood pressure by 2–10 mmHg (38). Long-term jogging practice is highly beneficial to alleviating hypertension, improving vital capacity and cardiac blood circulation (39), enhancing cardiopulmonary function, and preventing cardiovascular and cerebrovascular diseases (40). One study summarized the benefits of exercise training in patients with hypertension, and concluded that jogging could help reduce blood pressure based on evidence (41). Another study conducted a one-year jogging intervention on the older adult population, and concluded that jogging can reduce the heart rate, systolic blood pressure and diastolic blood pressure of the older adult (42). In our study, there was a slight change in cardiopulmonary function index after the intervention in the jogging group, systolic blood pressure was significantly reduced after the intervention. However, diastolic blood pressure did not change significantly, which might be due to the fact that the intervention time was not long enough. These results demonstrate that jogging for as short as 2 months still plays a positive role in improving cardiopulmonary function. Certainly, literatures suggest that if people want to effectively improve their physical fitness and cardiopulmonary function through simple physical activities, long-term persistence is indispensable.

Both jogging and rope skipping are systemic physical activities, and all muscles in major joints of the body are involved in the activities. In our study, jogging is significantly better than rope skipping in improving physical fitness and cardiopulmonary function through this short-term training. We consider the following factors that may cause the differential effects. First, the targeted muscle is different. Jogging can be generally decomposed into four stages: folding forward swing, pressing down to prepare for landing, landing slowly and stepping back (43). The movement process includes torsion of the trunk, forward tilt and rotation of the pelvis. The step size adjustment exercises the explosive force of leg muscles and the flexibility of hip joints. The step frequency adjustment exercises muscle contraction speed and neuromuscular coordination ability (44, 45). Therefore, running is more focused on the enhancement of lower limb muscles and hip muscles. In comparison, the muscle groups trained by rope skipping are more average (46). Jumping action mainly depends on the muscle contraction of calf, such as gastrocnemius muscle and soleus muscle, which can enhance the strength and endurance of calf. The core muscle groups in the abdomen and back are involved in maintaining the balance and stability of the body and can enhance core strength. The whole process of upper limb rope control depends on resistance control, which can be exercised to shoulder, biceps, triceps, and forearm muscles such as brachioradialis and pronator teres that control grip strength (47). Therefore, rope skipping focuses on comprehensively improving the strength and coordination of the muscles in all parts of the body. However, due to the short intervention time in our study, compared with rope skipping, jogging with more focused muscle emphasis may be more effective in certain performance evaluations such as long jump. Second, the arm muscles and shoulder joints act differently. The swing arm plays an important role in running because it helps reduce vertical oscillations, reverses vertical angular momentum of lower limbs, and minimizes the rotation of head, shoulders, and torso (48). Rope skipping is a fierce movement around the shoulder joint (49). The arm and shoulder joint action mode of running is closer to the arm extension action of the upper limb of the standing long jump, so as to assist jogging in improving standing long jump performance. Third, the nature of movement is different between these two. In the exercise requirements stipulated in this study, jogging belongs to continuous exercise, and respiratory muscles need to work continuously and steadily during it. Regularly continuous exercise may make respiratory muscles good exercise, increase the expansion and contraction ability of thoracic cavity, and improve vital capacity (50). Rope skipping belongs to intermittent exercise that can be either high-intensity or low-intensity. The process of high-intensity intermittent exercise requires rapid gas exchange in the lungs and accelerated beating of the heart to meet the energy supply needs of high-intensity exercise periods, which can indeed improve cardiopulmonary function (51). However, the rope skipping intervention in this study belongs to low-intensity intermittent exercise, and the short-term intervention effect seems significantly smaller than that of jogging. Fourth, sports emotions are different. Jogging is a sport that could be performed by many people at the same time. Influenced by the psychology of the competition or companion, the participants can be joyful and their mood can be easily in a higher state. Under a high mood, sympathetic nerve excitement takes advantage, metabolic rate in the body is enhanced, and blood sugar may be spiked, so that the energy supply system might work better. These effects could also delay the occurrence of fatigue, which is conducive to increasing the intensity of exercise and prolonging the time of exercise, so that respiratory muscles can be trained, vital capacity can be increased, and pulmonary ventilation function and heart pump capacity can be improved (52). Therefore, the changes of cardiopulmonary function in the jogging group could be more significant.

Our research results clearly show some positive effects of jogging on physical fitness and cardiopulmonary function, and to some extent, show that jogging is better than rope skipping in short-term training. This is of great significance for exercise intervention strategies in the field of public health and helps policy makers and health professionals better understand how to design effective and feasible exercise plans and recommendations to promote public health. For example, for young people, for those who want to improve their physical fitness and cardiopulmonary function in a short period of time, they may be more suitable to have regular low-intensity jogging or high-intensity rope skipping training. For groups who want to cultivate the habit of physical activity to maintain exercise for a long time, they may be more suitable to have long-term, regular, and low-intensity jogging or low-intensity rope skipping exercises. By understanding the effects and applicable groups of different sports, it is helpful to design more scientific and personalized sports intervention programs for not only children and adolescents, but also middle-aged and older adult people in the future, to promote public health more effectively and comprehensively.

Although our results demonstrate the importance and usefulness of physical activities especially jogging, there are some outcomes in our study that are inconsistent with our expectation or indicate that the intervention has not shown a significant effect. There was no significant difference in most physical test scores and cardiopulmonary function in the rope skipping group before and after the intervention. And the difference analysis of each performance in the jogging group, the rope skipping group and the control group showed no statistical difference between the groups. The *d* values of the jogging group, the rope skipping group and the control group were analyzed and showed no statistical difference between the groups except for the standing long jump. We believe that one possible reason is that the intensity of rope skipping is too low. Another possibility could be the selection of sport type, which is single and very limited that cannot fully cover the spectrum of various physical functions. In addition, the ways and methods of supervision in the intervention process are technically and practically limited, which could not ensure and guarantee that all participants completed the exercise requirements in high quality. Furthermore, the participants’ attitudes and behaviors toward the two physical tests before and after the intervention were different, which could also affect the test results.

Despite short-term physical activities are able to improve people’s physical fitness and cardiopulmonary function in stage, it is affected by various factors to cultivate long-term good physical activity habits (53–57), among which starting as early as possible and adhering to longer-term regular physical activities are essential and critical. One study revealed that habit formation interventions are effective in fostering physical activity habit (58). Our results demonstrated that there was no significant difference in the questionnaire scores half a year after the intervention among the three groups, and there was also no statistical difference in the *d* values (the questionnaire scores half a year after intervention minus the ones before the intervention) among the three groups, indicating that there was no difference in the effect of the two sports on the cultivation of physical activity habits, which suggest two-month intervention of either jogging or rope skipping may have little effect on cultivating college students’ good physical activity habits. Physical activity actually plays an important role in the formation of students’ good behavior habits (59). The reason for this result may be that the cultivation of good physical activity habits not only requires long-term exercise maintenance, but also needs to be accompanied by various factors such as objective environment, knowledge learning, personal cognition, and group relations. It seems certainly not enough to cultivate stable physical activity habits only through short-term intervention with single sport.

With significant impacts achieved from our short-term intervention of jogging and rope skipping, we realize that there are several limitations in our study. First, our study participants were recruited from a single university campus in China, which is limited in medicine-related majors that lacks broad student coverage. Second, female participants were higher than that of the males in the questionnaire survey and intervention study. We believe these factors may cause certain bias in the recruitment selection and result interpretation. In addition, our exercise intervention was primarily supervised by a software, in which we could only prevent some cheating behaviors from the research participants by checking cellphone screenshots and periodically exercise spot checking, but failed to make a conclusion of no any flaw or even cheating behavior.

## 5 Conclusion

Jogging have more positive significance for physical fitness and cardiopulmonary function maintenance and improvement. If one wants to improve physical fitness in a shorter period of time, then jog is preferable. Regarding jogging, it is recommended no less than four times a week and no less than 40 min each time. It is necessary to warm up fully before exercise, and the warm-up time is better to be 10–15 min. If the sports foundation is weak, the warm-up time could be extended appropriately to prevent injury. The formal jogging should not be less than 25–30 min, and the entire process should be uninterrupted. The exercise intensity is based on moderate intensity aerobic exercise, i.e., the major performance includes significantly fast breathing, obvious sweating, and heart rate increase to 140 beats/min, which can be gradually increased. Post-exercise stretching is also crucial that no less than 5 min. Our findings in this study are of great significance in providing clues and guidance for future studies in promoting physical health and fitness in college students. We hope in future research that increase in the intensity of physical activity, or use a combination of jogging and skipping should be conducted in-depth, or add other age subpopulations in the groups simultaneously to conduct research so that the public health intervention research benefit groups continue to expand.

## Data Availability

The raw data supporting the conclusions of this article will be made available by the authors, without undue reservation.
