# Supplementary material for: Differential impacts of jogging and rope skipping in college students in China based on physical test score: a randomized controlled trial baseline indicator comparison in the intervention
Source: Front Public Health. 2025 Apr 8;13:1570768. doi: 10.3389/fpubh.2025.1570768 (PMC12011860; doi:10.3389/fpubh.2025.1570768)
Supplement: Supplementary file 1 [file Data_Sheet_1.docx]

Table S1. Summary of physical activity suggestions for the participants in jogging, rope skipping and control groups

| Physical activity | Goal |
| --- | --- |
| Jogging group | |
| Warm-up preparation | ≥15 min |
| Running requirements |  |
| Male | ≥30 min |
| Female | ≥25 min |
| Intensity recommendation | Jogging at a constant speed. Exercise intensity is based on the standard of moderate-intensity aerobic exercise, which is mainly manifested as a significant increase in respiration, a significant sweating state, and an increase in heart rate to 140 / min |
| Posture recommendation | Uniform breathing, natural arm swing, two-step with one exhalation and three-step with one inhalation; to ensure a certain stride, body leans forward with physical and mental relaxation as basic criteria |
| Stretch | ≥5 min |
|  | |
| Rope skipping group | |
| Warm-up preparation | ≥15 min |
| Running requirements |  |
| Male | ≥30 min |
| Female | ≥25 min |
| Intensity recommendation | Rope skipping 3 min (70 ~ 80 times / min) as a cycle with a rest for 2 min interval for a total of 30 min (exercise intensity based on the standard of moderate intensity aerobic exercise, that is, the main performance is significantly fast breathing, obvious sweating state, and heart rate increase to 140 / min) |
| Posture recommendation | Single rope skipping. Chest raised, knee slightly bent, with front feet to take off and land buffered, arms close to the body to shake the arms with normal breathing during rope skipping |
| Stretch | ≥5 min |
|  | |
| Control group | |
|  | Maintain previous intensity of physical activity with no intentional or significant change |

Table S2. Changes of secondary outcomes before and after the intervention

| Group (n) | Project/Index | Prior intervention | Post intervention | Statistics (*t/Z*) | *p* | 95%*CI* |
| --- | --- | --- | --- | --- | --- | --- |
| Jogging (29) | Body composition score | 71.51±5.06 | 71.74±4.86 | 0.161 | 0.873 | [-2.79, 3.26] |
|  | BMI score | 97.93±6.20 | 97.24±7.02 | -0.571 | 0.573 | [-3.16, 1.79] |
|  | Sit-ups (female) or pull-ups (male) score | 64.59±29.93 | 60.24±27.53 | -1.933 | 0.063 | [-8.95, 0.26] |
|  | Standing long jump score | 72.00±14.32 | 76.45±11.92 | 3.048 | 0.005 | [-1.46, 7.44] |
|  | Sitting forward flexion score | 85.00 (16.50) | 78.00 (17.00) | -1.629 | 0.103 | [-5.77, 5.84] |
|  | Vital capacity score | 85.00 (22.00) | 90.00 (25.00) | -0.829 | 0.407 | [-2.15, 3.80] |
|  | 50 meters score | 73.86±12.49 | 72.17±11.35 | -0.876 | 0.389 | [-5.64, 2.26] |
|  | 800 (female) or 1,000 (male) meters score | 74.10±10.31 | 72.28±11.51 | -0.991 | 0.330 | [-5.61, -1.95] |
|  | Systolic blood pressure (mmHg) | 125.07±10.56 | 121.24±10.07 | 2.139 | 0.041 | [-7.49, -0.16] |
|  | Diastolic blood pressure (mmHg) | 67.66±7.61 | 66.93±8.66 | 0.402 | 0.691 | [-4.42, 2.97] |
|  | Heart rate (beat/min) | 79.03±9.65 | 80.14±11.07 | -0.437 | 0.665 | [-4.07, 6.27] |
|  | | | | | | |
| Rope skipping (29) | Body composition score | 71.75±4.05 | 72.39±4.46 | 0.540 | 0.593 | [-1.78, 3.06] |
|  | BMI score | 96.55±9.36 | 95.86±8.25 | -0.571 | 0.573 | [-3.16, 1.78] |
|  | Sit-ups (female) or pull-ups (male) score | 78.00 (57.50) | 72.00 (27.50) | -0.686 | 0.493 | [-4.86, 5.55] |
|  | Standing long jump score | 72.00 (14.00) | 72.00 (13.00) | -0.534 | 0.593 | [-5.76, 3.97] |
|  | Sitting forward flexion score | 78.00 (20.00) | 76.00 (14.00) | -1.261 | 0.207 | [-3.83, 0.38] |
|  | Vital capacity score | 85.00 (23.00) | 80.00 (19.00) | -1.151 | 0.250 | [-10.17, 1.55] |
|  | 50 meters score | 75.79±9.56 | 75.21±9.21 | -0.614 | 0.544 | [-2.54, 1.37] |
|  | 800 (female) or 1,000 (male) meters score | 76.00 (14.00) | 72.00 (12.50) | -2.521 | 0.012 | [-7.29, -0.71] |
|  | Systolic blood pressure (mmHg) | 125.07±9.43 | 128.00 (20.00) | -0.387 | 0.698 | [-3.21, 5.15] |
|  | Diastolic blood pressure (mmHg) | 68.17±6.79 | 67.10±7.54 | 0.568 | 0.574 | [-4.92, 2.78] |
|  | Heart rate (beat/min) | 84.07±15.31 | 82.21±13.05 | 0.924 | 0.363 | [-5.99, 2.27] |
|  | | | | | | |
| Control (34) | Body composition score | 73.31±5.53 | 71.43±4.65 | -1.366 | 0.181 | [-4.67, 0.92] |
|  | BMI score | 94.12±10.48 | 93.53±11.78 | -0.442 | 0.661 | [-3.30, 2.12] |
|  | Sit-ups (female) or pull-ups (male) score | 59.56±33.89 | 60.18±41.68 | 0.163 | 0.872 | [-7.11, 8.34] |
|  | Standing long jump score | 74.76±13.35 | 74.00±15.46 | -0.587 | 0.561 | [-3.42, 1.89] |
|  | Sitting forward flexion score | 80.15±10.60 | 78.15±11.08 | -1.893 | 0.067 | [-4.15, 0.15] |
|  | Vital capacity score | 100.00 (15.00) | 90.00 (20.00) | -1.630 | 0.103 | [-7.37, 2.32] |
|  | 50 meters score | 74.74±6.49 | 74.56±10.15 | -0.157 | 0.876 | [-2.46, 2.10] |
|  | 800 (female) or 1,000 (male) meters score | 76.00 (15.00) | 74.00 (25.00) | -2.983 | 0.003 | [-7.92, -1.73] |
|  | Systolic blood pressure (mmHg) | 121.24±11.84 | 119.29±11.84 | 0.979 | 0.335 | [-5.97, 2.09] |
|  | Diastolic blood pressure (mmHg) | 65.65±8.21 | 65.91±9.29 | -0.181 | 0.858 | [-2.71, 3.24] |
|  | Heart rate (beat/min) | 79.32±14.59 | 81.00±14.22 | -0.541 | 0.592 | [-4.63, 7.98] |

Table S3. Comparison of intervention effects between groups

| *d* | Control group (n=34) | Jogging group (n=29) | Rope skipping group (n=29) | Statistics (*F/H*) | *p* |
| --- | --- | --- | --- | --- | --- |
| Body composition score | -1.88±8.01 | 0.24±7.95 | 0.64±6.36 | 1.038 | 0.240 |
| BMI score | 0.00 (0.00) | 0.00 (0.00) | 0.00 (0.00) | 0.003 | 0.998 |
| Sit-ups (female) or pull-ups (male) score | 0.00 (12.75) | -4.34±12.10 | 0.00 (12.50) | 1.363 | 0.506 |
| Standing long jump score | 0.00 (6.50) | 2.00 (8.00) | 2.00 (10.50) | 6.161 | 0.046 |
| Sitting forward flexion score | -2.00 (5.00) | -2.00 (6.50) | 0.00 (5.50) | 0.815 | 0.665 |
| Vital capacity score | 0.00 (10.00) | 0.00 (5.50) | -2.03±9.84 | 4.497 | 0.106 |
| 50 meters score | 0.00 (6.50) | 0.00 (8.00) | -0.66±5.12 | 0.260 | 0.878 |
| 800 (female) or 1,000 (male) meters score | -4.00 (11.25) | 0.00 (8.00) | -2.00 (6.50) | 2.551 | 0.279 |
| Systolic blood pressure (mmHg) | -1.94±11.56 | -3.83±9.64 | 0.97±10.99 | 1.451 | 0.240 |
| Diastolic blood pressure (mmHg) | 0.26±8.54 | -0.72±9.71 | -1.07±10.13 | 0.172 | 0.842 |
| Heart rate (beat/min) | 1.68±18.07 | 1.10±13.59 | -1.86±10.85 | 0.504 | 0.606 |

Notes: *d*, the data after the intervention minus the ones before the intervention.

Table S4. Comparison of intervention effects of standing long jump between groups

| Group₁- Group₂ | Statistics (*H*) | *p* | 95%*CI* |
| --- | --- | --- | --- |
| Control group - jogging group | -2.460 | 0.014 | [-10.04, -0.39] |
| Control group - rope skipping group | -0.840 | 1.000 | [-4.69, 4.96] |
| Jogging group - rope skipping group | 1.559 | 0.119 | [0.33, 10.36] |

Table S5. Comparison of participants’ physical activity questionnaire scores

|  | Control group (n=34) | Jogging group (n=29) | Rope skipping group (n=29) | Statistics (*H*) | *p* | *η^2^* |
| --- | --- | --- | --- | --- | --- | --- |
| Prior intervention | 49.18±11.71 | 47.48±9.40 | 44.00 (18.00) | 1.029 | 0.598 | 0.102 |
| Post intervention | 50.18±11.58 | 53.24±11.26 | 54.76±12.28 | 1.266 | 0.287 | 0.024 |
| Post intervention half a year | 47.76±13.28 | 45.86±10.12 | 47.93±13.14 | 0.257 | 0.774 | 0.023 |
| *d* | -0.41±8.83 | -0.62±12.24 | 1.34±13.15 | 0.264 | 0.769 | 0.077 |

Notes: *d*, the data half a year after the intervention minus the ones before the intervention.

Table S6. Gender-stratified analysis of total score of body test after intervention

| Subgroup | n (%) | Jogging group | | Rope skipping group | | Control group | | Statistics (*F*) | *p* | *η^2^* |
| --- | --- | --- | --- | --- | --- | --- | --- | --- | --- | --- |
| Gender |  | Mean | SD | Mean | SD | Mean | SD |  |  |  |
| Male | 28 (30.4) | 78.12 | 6.57 | 73.66 | 9.85 | 70.06 | 8.75 | 2.004 | 0.156 | 0.138 |
| Female | 64 (69.6) | 78.33 | 6.78 | 77.62 | 8.68 | 81.02 | 9.31 | 1.031 | 0.363 | 0.033 |
| Total | 92 (100) | 78.27 | 6.60 | 76.25 | 9.13 | 78.12 | 10.28 | 0.473 | 0.625 | 0.011 |

Table S7. Sensitivity analyses of leading outcome at 2 months

| Model | Effect | Jogging group | | Rope skipping group | | Control group | | Statistics (*F/H*) | *p* | *η^2^* |
| --- | --- | --- | --- | --- | --- | --- | --- | --- | --- | --- |
|  |  | n | Total score of body test | n | Total score of body test | n | Total score of body test |  |  |  |
| Model 1 | Total | 34 | 78.91±6.62 | 34 | 75.66±9.57 | 34 | 78.12±10.28 | 1.214 | 0.301 | 0.024 |
|  | Subgroup 1 | 19 | 79.74±7.74 | 18 | 77.11±10.22 | 22 | 78.44±11.53 | 0.318 | 0.729 | 0.011 |
|  | Subgroup 2 | 15 | 77.86±4.91 | 16 | 74.04±8.82 | 12 | 77.54±7.91 | 1.246 | 0.298 | 0.059 |
| Model 2 | Total | 34 | 78.91±6.62 | 34 | 75.52±9.63 | 34 | 78.12±10.28 | 1.329 | 0.269 | 0.026 |
|  | Subgroup 1 | 19 | 79.74±7.74 | 18 | 76.94±10.41 | 22 | 78.44±11.53 | 0.356 | 0.702 | 0.013 |
|  | Subgroup 2 | 15 | 77.86±4.91 | 16 | 73.92±8.71 | 12 | 77.54±7.91 | 1.349 | 0.271 | 0.063 |
| Model 3 | Total | 34 | 78.77±6.54 | 34 | 75.66±9.57 | 34 | 78.12±10.28 | 1.138 | 0.325 | 0.022 |
|  | Subgroup 1 | 19 | 79.58±7.66 | 18 | 77.11±10.22 | 22 | 78.44±11.53 | 0.282 | 0.755 | 0.010 |
|  | Subgroup 2 | 15 | 77.73±4.83 | 16 | 74.04±8.82 | 12 | 77.54±7.91 | 1.199 | 0.312 | 0.057 |
| Model 1: All eight participants who were lost in follow-up were presumed to have a 1-point increase in the total scores of the physical test after the intervention  Subgroup 1: Below junior  Subgroup 2: Junior and above  Model 2: Those four participants in the jogging group who were lost were presumed to have a 1-point increase in the total scores of the physical test after the intervention  Model 3: Those four participants in the rope skipping group who were lost were presumed to have a 1-point increase in the total scores of the physical test after the intervention | | | | | | | | | | |

Figure S1. Study procedure


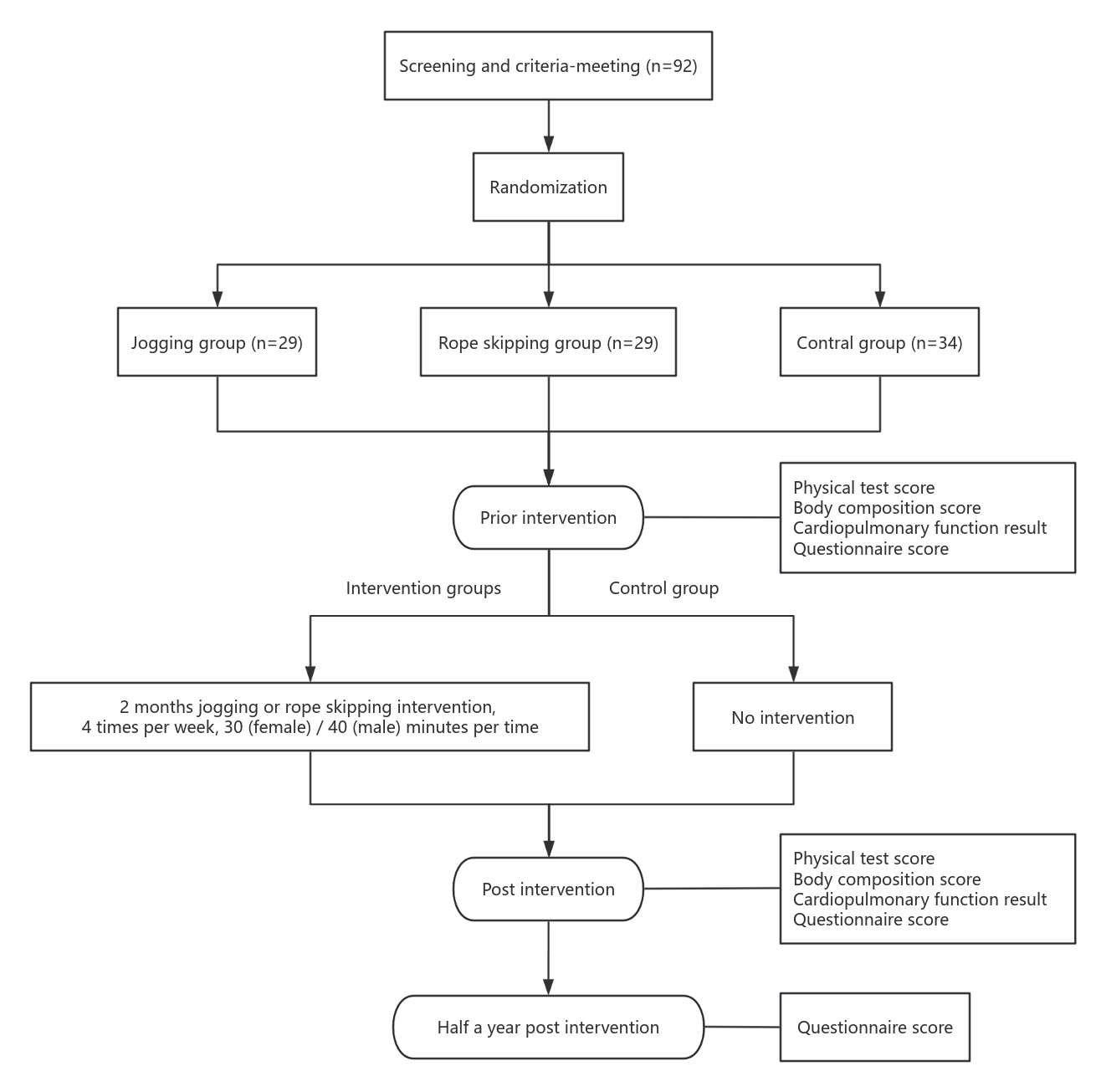


Figure S2. selection process flowchart


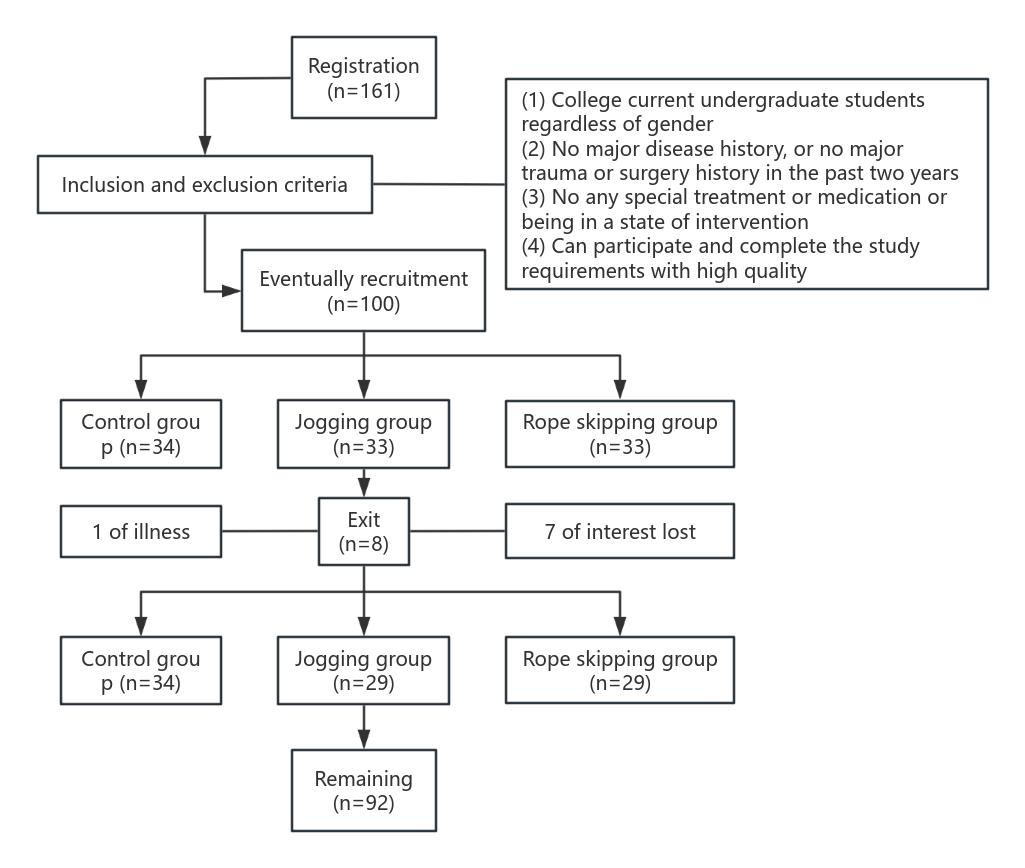


1. $\text{n}=\frac{\text{2（z}_{\alpha}+\text{z}_{\beta}\text{）}^{2}*\sigma^{2}}{\delta^{2}}$
